# Study protocol: a pragmatic randomised controlled trial of a 12-week physical activity and nutritional education program for overweight Aboriginal and Torres Strait Islander women

**DOI:** 10.1186/1471-2458-11-655

**Published:** 2011-08-19

**Authors:** Karla J Canuto, Robyn A McDermott, Margaret Cargo, Adrian J Esterman

**Affiliations:** 1 School of Health Sciences, University of South Australia, Adelaide, Australia; 2 Sansom Institute for Health Research, University of South Australia, Adelaide, Australia

## Abstract

**Background:**

Aboriginal and Torres Strait Islander women have a higher prevalence and incidence of obesity and type 2 diabetes than non-Indigenous Australian women. Physical inactivity is a key modifiable risk factor for obesity and evidence shows that even modest reductions in waist circumference (WC) have significant health benefits. Trialing physical activity programs in difficult-to-reach high risk groups, especially urban Indigenous Australians poses distinct implementation challenges.

**Methods/Design:**

The trial objective is to evaluate the effectiveness of a structured 12-week physical activity group program with nutritional advice. The design is a pragmatic randomised controlled trial. This study protocol describes the implementation and evaluation of the program. Participants are randomised into either an intervention or waitlisted group. The waitlisted group have a 12 month waiting period before commencing the 12-week program. Participant data is collected at baseline, 12, 24 and 52 weeks. Participants are Aboriginal and Torres Strait Islander women, aged 18-64 years with a waist circumference greater than 80 centimetres residing in Adelaide. The primary outcome measure is WC change immediately post program from baseline. Secondary outcomes include short term and long term changes in WC, weight, blood pressure, fasting blood glucose, insulin, insulin resistance (calculated HOMA), haemoglobin A1C (HbA1C), triglycerides and C-reactive protein (CRP). Behavioural and psychosocial surveys are administered to assess physical activity, dietary intake and the participant's motivation, self-efficacy and perceived social support for physical activity. Qualitative interviews focusing on participants' motivation, enablers and barriers to healthy eating and physical activity will be undertaken. Implementation fidelity and participation are also assessed.

**Discussion:**

The Aboriginal and Torres Strait Islander Women's Fitness Program (WFP) is designed to provide a rigorous physiological and client-based evaluation of a structured 12-week program aimed to increase metabolic fitness and reduce WC in this high risk population. Evaluation results aim to provide the support necessary to design programs that are accessible, affordable and effective at reducing WC, while also improving the metabolic profile of overweight Aboriginal and Torres Strait Islander women.

**Trial registration:**

Australian New Zealand Clinical Trials Registry ACTRN12610000224022

## Background

The Australian 2004-5 National Health Survey demonstrates that adult overweight and obesity is continuing to rise with 54% of non-Indigenous Australians classified as either overweight or obese compared with 60% of Aboriginal and Torres Strait Islander Australians [1]. There is a disparity among women with 45% of non-Indigenous women classified as overweight or obese compared to 58% of Aboriginal and Torres Strait Islander women. Overweight/obese Aboriginal and Torres Strait Islander women have a lower median age compared to non-Indigenous women (38 vs. 48 years) respectively [1]. Additionally the age-adjusted rate of diabetes for Aboriginal and Torres Strait Islander women are four times the rate for non-Indigenous Australian women [2].

It is established that both waist circumference (WC) and waist to hip ratio are significantly associated with increased risk of cardiovascular disease and that the distribution of body fat is the most important predictive risk factor for the development of type 2 diabetes mellitus (T2DM) [3-6]. The 2000 AusDiab study found that large WCs were associated with undiagnosed T2DM [7]. A 2004 study of Australian Aboriginal people in the Northern Territory found that WC tended to have better sensitivity and specificity in relation to predicting T2DM compared with body mass index (BMI) or waist to hip ratio [8].

Physical activity and diet have been shown to decrease the incidence of T2DM in 'at risk' participants [9] and assist those with T2DM to manage the condition and reduce complications [10,11]. In 2008, a systematic review assessed the effectiveness of randomised controlled clinical interventions designed to reduce the incidence of T2DM in participants at high risk. The review found that interventions combining exercise with diet decreased the incidence of T2DM in participants with impaired glucose tolerance (IGT) or metabolic syndrome [9]. Another review of clinical interventions evaluating the effectiveness of different modes of exercise on glucose control and risk markers for complications in patients with T2DM found small to moderate beneficial effects on glucose control (all modalities), small beneficial effects on some related risk factors for complications of diabetes and further small additional benefits from combining aerobic and resistance exercise [11]. The findings from clinical trials provide support for physical activity and nutrition interventions for people with T2DM or at high risk of developing T2DM.

The evidence supporting the effectiveness of interventions targeting high-risk Aboriginal populations in the community is less robust. It is well-established that implementing rigorously evaluated interventions in community settings is difficult [10,12]. A literature review of these community lifestyle interventions to prevent T2DM was published in 2003 [10]. Of the ten studies conducted in adult populations only one had a randomised design and six did not report any clinical markers in their results. However, many reported an increase in knowledge and physical activity among the intervention groups [10]. Similarly Clifford et al (2011) conducted a literature review of lifestyle interventions targeting Indigenous Australians [12]. Of the twenty studies identified, only four targeted nutrition and physical activity for Indigenous adults, three were conducted in remote areas and one with an urban Indigenous population. All four reported health outcomes and used validated measures; two of the studies were cohorts, one without a control and the other study had a self-selected control group, the third study was a pre/post design without a control group and the forth was a time series design with a randomised control group [12]. One of those reviewed was the Looma Healthy Lifestyle program in Western Australia which focused on education, health promotion and organised physical activities. The program was evaluated using a pre/post design which found initial positive results among a high risk cohort, however weight loss among the participants was not sustained [13]. None of the programs included a structured, evidence-based physical activity program facilitated by qualified and trained instructors. Only one of four studies reported a quantitative measure of program exposure and no study provided a logic model to delineate how the intervention would lead to the primary and secondary outcomes. Without this information it is difficult to rule out implementation failure or theory failure in interpreting the results [12]. Clifford et al (2011) concluded that intervention studies that target healthy lifestyles in Indigenous populations are undertaken infrequently, and are generally of poor methodological quality. Satterfield's (2003) review also concluded that there is a need for community-based interventions to use strong research designs.

Although limited, some small-scale programs implemented in a community setting for high-risk Aboriginal populations have shown encouraging results. In New Zealand, a four month lifestyle intervention was conducted with Maori participants at high risk of developing T2DM [14]. The program consisted of fortnightly clinic visits, nutrition and cooking workshop and individualised exercise programs that prescribed five, 30 minute sessions per week. The program also offered group exercise sessions [15]. The program demonstrated positive results (after the intervention compared to baseline) in most risk markers such as weight loss(3.1 kg), reduction in waist circumference (7.0 cm), body fat (2.0 kg), insulin sensitivity, fasting glucose (0.5 mmol/L) and fasting insulin (5.1 mIU/L)[14].

In 2007, an eight week physical activity program called the Women's Fitness Challenge (WFC) was implemented on Thursday Island in the Torres Strait, which was facilitated by a qualified Torres Strait Islander woman [16]. Participants attended structured aqua aerobics and group circuit class and were assigned pedometers to record their daily steps. Classes included graded exercises to accommodate overweight women, older women and those who had not participated in these types of activities before. However, they were challenging enough for younger and fitter participants with experience in group exercise classes. Participants who attended a final assessment for the program (n = 10) demonstrated a mean reduction in WC of 4.93 cm. There was a strong correlation between the number of self recorded steps (using a pedometer) and reduced WC (R squared = 0.56) and a moderate correlation between the number of classes attended and reduction in WC (R squared = 0.42) [16]. The eight-week WFC informed the development of the Aboriginal and Torres Strait Island Women's Fitness Program.

In summary, evidence for effectiveness of obesity reduction programs implemented in a community setting is mixed. Those reporting success appear to have the following characteristics [17-19]:

• They emphasise physical activity and diet, rather than diet alone

• They accommodate the preferences of participants

• They are conducted in groups

• They have varied and progressive levels and types of activities e.g. combined aerobic and resistance training

In addition, for programs to be sustained, they should be: 1) culturally acceptable; 2) feasible and located close to work or home; 3) easily incorporated into daily schedules, including family activities; and 4) able to show goal attainment which is realistic and appropriate. Compared to the costs to the individual, the health care system and the community, prevention at any level is a highly cost-effective strategy [20].

## Methods/Design

### Study aims

The primary aim of this study is to evaluate whether a structured 12-week physical activity group program with nutritional advice can reduce central obesity (as measured by waist circumference) among Aboriginal and Torres Strait Islander women in an urban setting. The secondary aim is to explore whether WC loss is maintained at 24 and 52 weeks. Additional aims include assessing changes in metabolic profile, participants' quality of life, their dietary and physical activity habits and their motivation, perceived support and self efficacy towards exercise. Qualitative interviews will supply feedback on the program, will help contextualise the quantitative data, explore the participants' motivation, enablers and barriers to healthy eating and physical activity and provide information about any co-interventions, contamination or unexpected/unintended consequences of the program.

### Study design & setting

The effectiveness of the WFP on the WC and metabolic profiles of Aboriginal and Torres Strait Islander women is being assessed using a pragmatic randomised controlled trial. The BMJ 'Clinical Evidence Glossary' defines a pragmatic study as 'an RCT designed to provide results that are directly applicable to normal practice...'[21]. There are many characteristics of a pragmatic RCT that differ from a clinical RCT such as lack of blinding, it may not employ placebos and it may focus on groups with a wide range of diagnoses [22]. The CONSORT guidelines [23] and the recommendations for pragmatic trials have been considered in the projects' design and methodology [24].

It is important that the study maintain methodological rigour whilst being culturally appropriate and ensuring that participants find the protocol acceptable. It was determined by the research team and the local Advisory committee that using traditional 'control groups' would not be acceptable. It was therefore decided that eligible participants would be randomly assigned to either the 'active' group who start the WFP immediately after their baseline assessment or to the 'waitlisted' group, who act as a comparison group for 12 months before 'crossing over' to participate in the program. Each wave of participants was randomised using the PEPI randomisation procedure to create two groups of equal size.

### Participants

Informed written consent was provided by all participants prior to baseline assessments. A copy of a plain English statement describing the research project is held by participants. The consent form includes information about withdrawal of consent and that withdrawal from the research project will not affect their status now or in the future.

### Inclusion criteria

1. Aged 18 - 64 years at recruitment;

2. Identify as Aboriginal and/or Torres Strait Islander;

3. Waist circumference > 80.0 cm at baseline assessment.

### Exclusion criteria

1. Pregnant at baseline assessment or if they become pregnant during the study period;

2. Physically unable to participate in the program based on medical advice from their treating doctor.

### Recruitment

Participants were recruited in three intakes (April 2010, September 2010 and March 2011). The first two programs were delivered at the City East Campus of the University of South Australia, Adelaide. The third program was based in an outer suburb (30 km from the Adelaide CBD) with a high proportion of Aboriginal residents.

Project staff visited local Aboriginal and Torres Strait Islander organisations, health centres and community groups to inform them of the project and ask them to display posters and distribute brochures. Several information sessions were held during each recruitment phase to allow potential participants to meet project staff and become fully informed on the project before formally registering to participate. A project flier distributed via e-mail proved to be an effective way of recruiting participants. In addition social networks were also an effective recruitment tool.

### Ethics

The study was approved by the University of South Australia's Human Research Ethics Committee and the Aboriginal Health Council of South Australia's Human Research Ethics Committee.

### Local Advisory Committee

The project is guided by an advisory committee. The committee was purposively formed for the project and is made up of local Aboriginal and Torres Strait Islander women. Some of members are representatives from the collaborating organisation Nunkuwarrin Yunti of South Australia Inc. The committee provides advice on feasibility of the project's procedures and assessments ensuring that all aspects of the project are culturally appropriate. The committee assisted in the adaptation of some surveys, gave advice on the questionnaires and will be invited to provide input into the interpretation of qualitative data.

### Staff

Staffing consists of three chief investigators, an associate investigator, and a Torres Strait Islander and Doctor of Philosophy candidate (KC). The project also employs a project coordinator, qualified fitness instructors, anthropometric assessors (ISAK accredited), a dietician and an experienced Aboriginal interviewer. All staff involved in delivering the project are female, which is essential to ensure cultural safety and sensitivity for our participants.

### Analysing the environmental determinants of obesity

The original project plan included an analysis of environmental determinant of obesity using a framework known as ANGELO (Analysis Grid for Environments Linked to Obesity). ANGELO is a process in which community members and key stakeholders attend workshops to indentify barriers to physical activity and healthy eating then formulate strategies to address the identified barriers. Prior to participants being recruited for this project, the Advisory Committee did a half day workshop to trial this framework. It was found not to be a useful tool in this setting within in the scope of the project.

### Program

The WFP is a structured 12-week physical activity group program with nutritional advice. The program consists of four key components.

1) 12 weeks of structured group exercise classes.

Each 45 - 60 minute exercise class includes a combination of aerobic and resistance training. Classes progressively increase in difficulty and intensity as the participants' fitness, strength and confidence increase. The program aims to have participants exercise at moderate to high intensity for at least 30 minutes per session. Exercises are graded according to level of difficulty to enable and challenge participants of all ability levels. Classes are coordinated and supervised by project staff and delivered by qualified instructors. The exercise classes are implemented as outlined in the 'Program Manual'. The Program Manual provides the instructors and staff with a set of guidelines and resources for implementing each exercise session.

2) Incidental activity and walking.

Participants are encouraged to reach 10,000 steps daily or 70,000 weekly. Pedometer readings are self-recorded in their 12-week exercise diaries. Staff document the recorded steps when the participants bring their exercise diaries to group sessions. The exercise diaries are also collected at the end of the program.

3) Nutrition Workshops

Four nutrition workshops led by a qualified dietician are conducted during the 12-week program. Topics include basic nutrition, food label reading, recipe modification and healthy takeaways options. The workshops highlight practical changes that the women can incorporate into their daily lives. Workshop content was adapted from the Whyalla Nutrition and Exercise Study [25] with permission from the principal researcher and author. The final workshop is an interactive cooking demonstration involving participants making three tasty, nutritious dishes.

4) Positive reinforcement and encouragement

Fortnightly newsletters are distributed to participants during the 12-week program. Each newsletter contains a healthy recipe, tips on nutrition and physical activity and acknowledges the participants who have logged the most steps for the fortnight. The project staff provide regular participant feedback on the quality of their exercise technique and their progress, as well as encouragement to wear their pedometer and record their daily steps.

The group exercise classes and nutrition workshops aim to bring together 'like minded' Aboriginal and Torres Strait Islander women facing similar barriers to living a healthy lifestyle, that harbours social support and creates a culturally 'safe' environment.

Waitlisted participants they also receive a pedometer, exercise diary. They also are posted monthly newsletters and are invited to attend workshops over the 12 month waiting period. The waitlisted participants are invited to suggest topics for these workshops. Some workshops that have been delivered based on participant requests include "Fad diets - myths and facts", "Healthy Recipes" and "How to stay healthy over Christmas".

### Program logic model

**Figure 1:**
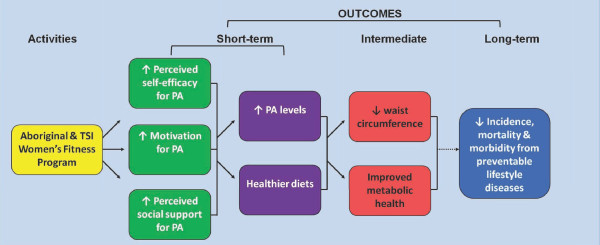
**Program logic model**. This abridged version of the program logic model depicts the predicted short-term, intermediate and long-term outcomes of the program.

Figure 1 shows an abridged version of the program logic model. The model depicts the anticipated change process through women's participation in the program. The 12-week WFP hopes to activate positive psychosocial (i.e. perceived self-efficacy, motivation and perceived social support) and behavioural changes (an increase in moderate and vigorous physical activity, reduced sedentary activity, reduced saturated fat intake, reduced sweetened drinks, increase in dietary fibre, fruit and vegetables intake) in the participants. These results should lead to intermediate outcomes of reduced WC and improved metabolic health. Long-term outcomes are beyond the scope of the research project however long-term follow up studies such as the Finnish Diabetes Prevention Program and Da Qing in China suggest that their participants had a reduction in the incidence of lifestyle chronic diseases for several years post program [26,27]. If the Aboriginal and Torres Strait Islander Women's Fitness Program produces results similar to the above mentioned studies, then it could be extrapolated that this would lead to a reduction in morbidity and mortality.

### Outcome Measures

Once a participant's registration form and consent is received their clinical and anthropometric assessments are taken. Participants are then given surveys to complete and a pathology request form for a fasting blood sample to be taken at their convenience. Written approval from a registered medical practitioner is required if a participant identifies a previous diagnoses of diabetes, hypertension, hyperlipidia, a heart condition, kidney disease or any other condition for which exercise may be contraindicated such as cancer. Participant requiring a medical clearance are given a letter to take to their GP which has a brief explanation of the program and an attachment for them to return. All participants have to option of requesting that their pathology results be forwarded to their GP for their medical record.

**Figure 2:**
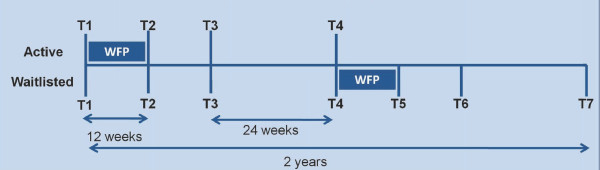
**Program timeline**. The program timeline is a depiction of the assessment time points (T1-T7) and the 12-week WFP for both the active and waitlisted groups.

Assessments are taken at baseline, 12, 24 and 52 weeks. Waitlisted participants are invited to participate in the same assessments at the same time as the active group, with additional follow up after they participate in the 12-week program. The program timeline (Figure 2) depicts the assessment time points for both groups.

Anthropometric assessments are taken by staff accredited by the International Society for the Advancement of Kinanthropometry (ISAK) using the International Standards for Anthropometric Assessment procedures [28]. ISAK assessors have an intra-tester technical error of less than 1.5% and an inter-tester reliability error of less than 2%.

i) Blood pressure and anthropometric assessments:

a. Blood pressure is measured using an Ormon T8 with an upper arm cuff after the participant has been seated for a minimum of 5 minutes. Three readings are taken and the mean of the second and third readings is also recorded.

b. Height is measured using a portable stadiometer (Leicester). The stretched stature is assessed twice to the nearest 0.1 cm; both measures and the average are recorded.

c. Weight is measured using portable digital scales (Tanita BC-532). Participants are asked to empty pockets and remove footwear and weighed wearing light clothing. Two measures and are taken to the nearest 0.1 kg. Both measurements and the average are recorded.

d. Waist and hip circumference are measured over light clothing to the nearest 0.1 cm using a flexible steel measuring tape (Lufkin w606 pm). Both measures and the average are recorded.

ii) Clinical assessments:

Venous blood samples are collected following a minimum 8 hour fast by accredited pathology services in the Adelaide metropolitan area. Pathology results are reported back to participants using the Health Scope Pathology Biochemistry Departments reference ranges [29].

a. Fasting blood glucose is analysed using Hexokinase assay with Siemens Advia 2400.

b. Fasting serum insulin is analysed using Chemiluminescent Immunoassay with Siemens Advia 2400.

c. HbA1C is analysed using Immunoturbidimetry assay with Roche Integra 800.

d. Total cholesterol, high-density lipoprotein (HDL), low-density lipoprotein (LDL) and triglycerides were analysed using Chemiluminescent Immunoassay with Siemens Advia 2400. LDL/HDL and Cholesterol/HDL ratios are also calculated.

e. C-reactive protein (CRP) is analysed using Immunoturbidimetry assay with Siemens Advia 2400.

f. Homeostatic Model Assessment (HOMA) is calculated using the formula;

$$\text{HOMA }=\;\left(\text{fasting glucose }\left[\text{mmol}∕\text{L}\right]\;\times \text{ fasting insulin }\left[\mu \text{U}∕\text{mL}\right]\right)∕\text{22}.\text{5}.$$

### Surveys

Participants are asked to complete psychosocial and self-report behaviour surveys in their own time. The following surveys are conducted at each assessment time point i.e. baseline, 12 weeks, 24 weeks and 52 weeks.

a. Current physical activity is assessed using the Sallis seven-day physical activity recall survey [30], modified to include time spent watching television.

b. The Victorian Cancer Council Food Frequency survey (DQESv2) is used to assess the participant's dietary intake [31] and fluid intake is assessed using an instrument designed for the program.

c. The SF-36 survey is used to assess perceived quality of life [32].

d. Perceived social support for physical activity is assessed using the 'Social Support and Exercise measure developed by Sallis et al [33]. This 12-item measure assesses perceived social support by friends and family separately. Each item is scored on a 5-point Likert scale. The measure has a Cronbach alpha range of 0.60-0.91 [34].

e. Perceived self-efficacy for physical activity is assessed using a measure developed by Sallis et al. This 12-item measure assesses two sub-dimensions: a) sticking to it; and b) making time for exercise. Each item is scored on a 5-point Likert scale [35]. The measure has a Cronbach alpha range of 0.83-0.85 [36].

f. The 'Motives for Physical Activities Measure' developed by Fredrick and Ryan REF assesses the strength of five motives for participating in physical activities: 1) interest/enjoyment 2) competence 3) appearance 4) fitness and 5) social. Each item is scored on a 5-point Likert scale. The measure has a Cronbach alpha range of 0.78-0.92 [37].

Each participant receives the results of selected assessments in personalised written form. This report includes anthropometric and pathology assessments and a summary of their 7 day physical activity survey and comments from the chief investigator/project GP. Participants can also opt to have a copy of their pathology results forwarded to their treating doctor.

### Process evaluation

The process evaluation assesses program dose and implementation fidelity. Program dose is measured in two ways. Project staff record participant attendance at each group exercise session and nutritional workshop. A continuous measure of dose is computed, expressed as total hours attended. Participants are provided a Yamax SW-200 pedometer to self-monitor and record their daily steps. Participants are asked to bring their exercise diaries to classes so these steps can be recorded by the project staff. The Yamax pedometers have been chosen as it is one of the most accurate and commonly used in research [38]. A continuous measure of steps is computed. This data will enable interpretation of program outcomes, with the aim of strengthening the study's internal validity.

Implementation fidelity of the group classes are assessed using a standardised form. A project officer in attendance at each session rates the extent to which each class is implemented as planned, noting any variations or purposeful adaptations. The implementation questions are scored on a 5-point scale. Participant and environmental factors that may influence the implementation of a session (positively or negatively) are also recorded. The fitness instructor who facilitated the class completes an implementation log which records their perceived enthusiasm of the participants and the overall success of the session. Comments on any participant or environment factors that influenced the class and general feedback on the session are also recorded on the implementation log form.

### Qualitative evaluation

At T2, 45 minute semi-structured one-on-one interviews are conducted with the aim of understanding individual, interpersonal and environmental factors that may influence participant engagement in the WFP. Waitlisted participants are also invited for an interview to assess their motivation to join the project, to gauge their current physical activity level compared to before they joined and if they have made any other changes to physical activity, diet or medication. At T3, participants who participated in the program will be purposively sampled based on their waist circumference trajectory from T1 to T2 and T3. The purpose of these interviews is to gain information on their current activity levels, their capacity to integrate physical activity into their lives following program completion and the motivators, enablers and barriers related to the maintenance of their waist loss or regain of centimetres that were previously lost. The interviews will help explain the quantitative outcomes and identify unintended consequences of the program.

### Sample Size Estimation

The primary outcome for the study is to assess if waist circumference reduction immediately after the program compared to baseline will be greater in the active group than the waitlisted group. Feasibility of this program has been demonstrated in a pilot program (WFC) in 2007. The eight week pilot program enrolled 24 women, 10 of whom had baseline and follow-up assessments (there was no comparison group). Those assessed lost a mean of 4.93 cm (95% confidence interval 2.44-7.41, SD = 3.47 p = 0.002) of WC from baseline (mean at baseline 91 cm to 86 cm at 8 weeks). The degree of waist loss was proportional to adherence to the program (r = 0.647) [39]. Therefore we assume an expected program effect of a reduction of 5 cm in WC after 12 weeks in the active group. The waitlisted group receive a modest intervention and are not strictly a control group therefore we estimate that the waitlisted group may have a WC reduction of 1 cm over the same time period equating to a 4 cm difference between groups. A sample size of 13 per group would have 80% power to detect a difference in waist circumference of 4 cm assuming a standard deviation of waist circumference of 3.47 cm and using a two sided independent samples t-test with a 0.05 level of significance. Experience from the WFC however predicts that nearly 60% of participants will be lost to follow-up which would increase the group size to 32. This calculation assumes that the participants are independent however many women will be colleagues or family members so we have increased our sample size estimation to 40 participants per group.

### Statistical Analysis

The active and waitlisted groups will first be compared at baseline (T1). Continuous variables will be compared using Student's T tests. Categorical variables will be compared using Chi-squared tests. No attempt will be made to adjust for clustering. Analyses will be undertaken using SPSS 16 and Stata 10.

The primary outcome measure (change in waist circumference between T1 and T2) will be compared between groups at T2 both crude (unadjusted) and adjusted for baseline waist circumference using analysis of covariance. Further analyses will adjust for other potentially confounding variables, such as age, number of children, education, employment status, household income and illness/disease.

Change in waist circumference between T1 and T3, and between T1 and T4 will be compared between groups both crude (unadjusted) and adjusted for baseline waist circumference. Further analyses will adjust for other confounding variables. The additional anthropometric and clinical assessments will be analysed in the same way to compare the active group with the waitlisted group at each time point.

Changes in major outcome measures over time will be explored using linear mixed modelling. Predictors of WC change, and possibly other measures, will be explored within the active group to detect any trends that may suggest barriers or enablers to positive change.

Further regression analyses will explore any association of psychological and behavioural variables with reduction in waist circumference.

The waitlisted participants will appear in the data set more than once as they 'cross over' to join the active group after their 12 months in the waitlisted group. This will be allowed for in the analysis using linear mixed modelling.

### Qualitative Interviews

All interviews will be digitally-recorded and transcribed by a professional transcriptionist to capture participant experiences. A social-ecological framework will be used to guide the analysis; analytic techniques will be derived from Miles and Huberman [40]. Interviews will be managed using QDA Miner. To enhance the credibility of the qualitative component, member checking, peer debriefing will be used [41].

## Discussion

The Aboriginal and Torres Strait Islander Women's Fitness Program is designed to provide a rigorous physiological and client-based evaluation of a structured 12-week group exercise and nutrition program (WFP) that can be implemented in a community setting. The WFP aims to improve the metabolic profile and reduce WC in this high risk population. The participants are followed up after the program to assess the short-term effectiveness of the program.

The project has encountered some challenging operational issues in mounting this pragmatic controlled trial. The original proposal aimed to implement the WFP in the Torres Straits, located in the far north of Australia. Allocated project funding did not make allowances to accommodate staff in the Torres Straits or support transportation for the research staff in the two sites for regular meetings. After lengthy negotiations the partners in the Torres Straits were unable to provide sufficient in-kind support to implement the project. The program therefore had to be adapted to accommodate Aboriginal women living in an urban setting, partnerships had to be established, and the processes of obtaining ethics and promoting the project to the urban community had to recommence.

Evaluation results will provide the support necessary to design programs that are accessible, affordable and effective at reducing WC and improving the metabolic profile of overweight Aboriginal and Torres Strait Islander women.

## Competing interests

The authors declare that they have no competing interests.

## Authors' contributions

KJC developed the 12-week program, contributed to the study design and is involved in the data collection and delivery of the project. KJC was primarily responsible for drafting the manuscript under the supervision of RAM and MC. RM developed the study design and is the holder of the NHMRC funding. MC and AJE contributed to the study design. AJE also provided statistical advice. All authors read and approved the final manuscript.

## Authors' information

Karla Canuto is an Indigenous PhD candidate. Karla's qualifications include a Bachelor of Sports & Exercise Science and a Graduate Diploma in Indigenous Health Promotion. Robyn McDermott is a Professor of Public Health in the Sansom Institute for Health Research. Professor McDermott is also a medical epidemiologist and public health physician with extensive experience and research history in lifestyle chronic disease in Aboriginal and Torres Strait Islander communities. Dr Margaret Cargo has expertise in theory-driven evaluation and implementation science. Dr Cargo has experience working with Aboriginal populations in community-based programs. Professor Adrian Esterman is a biostatistian and epidemiologist.

## Pre-publication history

The pre-publication history for this paper can be accessed here:

http://www.biomedcentral.com/1471-2458/11/655/prepub
